# Supplementary material for: Molecular phylogeny and diversification timing of the Nemouridae family (Insecta, Plecoptera) in the Japanese Archipelago
Source: PLoS One. 2019 Jan 11;14(1):e0210269. doi: 10.1371/journal.pone.0210269 (PMC6329508; doi:10.1371/journal.pone.0210269)
Supplement: S1 Table — Numbers of individuals (N), presence of male (M), female (F) and imago (im), DNA-species delimitation (GMYC). (DOCX) [file pone.0210269.s001.docx]

**S1 Table**. Location information of samples of East Asia Nemouridae. Numbers of individuals (N), presences of male (M), female (F) and imago (im), DNA-species delimitation (GMYC).

| Genera | Species | n | M | F | im | Country | Site | Latitud | Longuitud | Altitude (m) | GMYC |
| --- | --- | --- | --- | --- | --- | --- | --- | --- | --- | --- | --- |
|  | A. bulla | 1 | x |  |  | Japan | Jikibamachi, Ehime | N33°51.579’ | E132°49.963’ | 280 | 1 |
|  |  | 1 |  | x |  | Japan | Shukunomachi, Ehime | N33°52.165’ | E132°49.914’ | 130 | 1 |
|  |  | 3 | x |  |  | Japan | Toyonakacho, Kagawa | N34°09.130’ | E133°40.277’ | 125 | 1 |
|  |  |  |  | x |  |  |  |  |  |  | 1 |
|  |  |  |  |  | x |  |  |  |  |  | 1 |
|  | A. decemseta | 1 |  | x |  | Japan | Saijo, Ehime | N33°45.322’ | E133°09.436’ | 1450 | 2 |
|  |  | 1 |  | x |  | Japan | Nomuracho Onogahara, Ehime | N33°28.508’ | E132°53.132’ | 1170 | 2 |
|  |  | 1 |  | x |  | Japan | Aonamimachi, Ehime | N33°52.825’ | E132°51.350’ | 330 | 2 |
|  |  | 1 |  | x |  | Japan | Shukunomachi, Ehime | N33°52.627’ | E132°50.463’ | 230 | 2 |
|  |  | 1 | x |  |  | Japan | Mt. Kasatori, Ehime | N33°34.011’ | E132°56.012’ | 1425 | 2 |
|  |  | 2 | x |  |  | Japan | Nomuratani River, Tokushima | N34°05.891’ | E134°04.187’ | 260 | 2 |
|  |  |  |  | x |  |  |  |  |  |  | 2 |
|  |  | 2 | x |  |  | Japan | Shoji, Kagawa | N34°09.974’ | E133°40.490’ | 85 | 2 |
|  |  |  |  | x |  |  |  |  |  |  | 2 |
|  |  | 2 | x |  |  | Japan | Yu River, Kagoshima | N31°12.255’ | E130°49.148’ | 105 | 2 |
|  |  |  |  | x |  |  |  |  |  |  | 2 |
|  |  | 2 | x |  |  | Japan | Tsukuyone, Tottori | N35°21.811’ | E134°29.662’ | 910 | 3 |
|  |  |  |  | x |  |  |  |  |  |  | 3 |
|  |  | 1 | x |  |  | Japan | Tsukuyone, Tottori | N35°21.811’ | E134°29.662’ | 910 | 3 |
|  |  | 3 | x |  |  | Japan | Judzuru-wa-ikoi Park, Hyogo | N34°14.880’ | E134°48.334’ | 200 | 3 |
|  |  |  | x |  |  |  |  |  |  |  | 3 |
|  |  |  |  | x |  |  |  |  |  |  | 3 |
|  |  | 2 | x |  |  | Japan | Joganji River, Toyama | N36°34.864’ | E137°26.463’ | 450 | 3 |
|  |  |  |  | x |  |  |  |  |  |  | 3 |
|  |  | 2 | x |  |  | Japan | Takagamine, Kyoto | N35°03.369’ | E135°43.440’ | 150 | 3 |
|  |  |  |  | x |  |  |  |  |  |  | 3 |
|  |  | 1 |  | x |  | Japan | Ibi River, Gifu | N35°31.885’ | E136°30.743’ | 105 | 3 |
|  |  | 1 | x |  |  | Japan | Lake Biwa, Shiga | N35°28.051’ | E136°06.479’ | 85 | 3 |
|  |  | 1 |  | x |  | Japan | Bandai-Asahi NP, Yamagata | N37°45.102’ | E140°05.857’ | 1315 | 4 |
|  |  | 2 |  | x |  | Japan | Mt. Azuma, Fukushima | N37°45.252’ | E140°18.221’ | 815 | 4 |
|  |  |  |  | x |  |  |  |  |  |  | 4 |
|  |  | 2 | x |  |  | Japan | Shoya, Nagano | N36°13.665’ | E138°36.299’ | 980 | 4 |
|  |  |  |  | x |  |  |  |  |  |  | 4 |
|  |  | 2 | x |  |  | Japan | Seki River, Niigata | N36°50.910’ | E138°08.958’ | 725 | 4 |
|  |  |  |  | x |  |  |  |  |  |  | 4 |
|  | A. dentifera | 2 | x |  |  | Japan | Shirahone, Nagano | N36°09.049’ | E137°37.704’ | 1350 | 5 |
|  |  |  |  |  | x |  |  |  |  |  | 5 |
|  |  | 1 | x |  |  | Japan | Myogatani River, Ehime | N33°32.922’ | E132°56.904’ | 900 | 5 |
| Amphinemura | A. flavostigma | 1 |  | x |  | Japan | Karakai Falls, Ehime | N33°44.912’ | E132°58.897’ | 870 | 6 |
|  |  | 2 | x |  |  | Japan | Myogatani River, Ehime | N33°32.922’ | E132°56.904’ | 900 | 6 |
|  |  |  | x |  |  |  |  |  |  |  | 6 |
|  | A. hainana | 1 |  | x |  | China | The Skytree, Mengla, Yunnan | N21°28’ | E101°35’ | 700 | 7 |
|  | A. hamiornata | 2 | x |  |  | China | Shiwandashan, Pearl River, Guangxi | N21°54.122’ | E107°54.292’ | 315 | 8 |
|  |  |  |  | x |  |  |  |  |  |  | 8 |
|  | A. longispina | 1 | x |  |  | Japan | Mt. Kasatori, Ehime | N33°33.600’ | E132°56.253’ | 1335 | 9 |
|  |  | 2 | x |  |  | Japan | Shikoku Karst, Ehime | N33°28.786’ | E132°55.105’ | 935 | 9 |
|  |  |  |  | x |  |  |  |  |  |  | 9 |
|  |  | 1 |  | x |  | Japan | Nishi-Aduma Sky Valley, Yamagata | N37°45.417’ | E140°06.199’ | 1205 | 10 |
|  |  | 1 |  | x |  | Japan | Nishi-Aduma Sky Valley, Yamagata | N37°45.102’ | E140°05.857’ | 1315 | 10 |
|  | A. megaloba | 1 | x |  |  | Japan | Shoya, Nagano | N36°13.665’ | E138°36.299’ | 980 | 11 |
|  |  | 1 |  | x |  | Japan | Saku, Nagano | N36°13.665’ | E138°36.299’ | 980 | 11 |
|  |  | 3 | x |  |  | Japan | Mt. Kasatori, Ehime | N33°33.979’ | E132°56.136’ | 1390 | 12 |
|  |  |  | x |  |  |  |  |  |  |  | 12 |
|  |  |  |  | x |  |  |  |  |  |  | 12 |
|  |  | 2 | x |  |  | Japan | Yada River, Hyogo | N35°24.518’ | E134°30.408’ | 420 | 12 |
|  |  |  |  | x |  |  |  |  |  |  | 12 |
|  | A. zonata | 1 | x |  |  | Japan | Yu River, Kagoshima | N31°11.994’ | E130°49.510’ | 150 | 13 |
|  |  | 1 | x |  |  | Japan | Fukumi-gawa, Ehime | N33°50.952’ | E132°52.112’ | 515 | 13 |
|  |  | 2 | x |  |  | Japan | Lake Biwa, Shiga | N35°28.051’ | E136°06.479’ | 85 | 14 |
|  |  |  |  | x |  |  |  |  |  |  | 14 |
|  | A. sp. n. | 3 | x |  |  | Japan | Mt. Kasatori, Ehime | N33°34.011’ | E132°56.012’ | 1425 | 15 |
|  |  |  | x |  |  |  |  |  |  |  | 15 |
|  |  |  |  | x |  |  |  |  |  |  | 15 |
|  |  | 1 |  | x |  | Japan | Tsukuyone, Tottori | N35°21.811’ | E134°29.662’ | 910 | 15 |
|  | A. sp. n. 1 | 2 | x |  |  | China | Damingshan, Guangxi | N23°30.373’ | E108°26.141’ | 1150 | 16 |
|  |  |  |  | x |  |  |  |  |  |  | 16 |
|  | I. nohirae | 2 |  | x |  | Japan | Kawanouchi, Ehime | N33°45.796 | E132°58.850’ | 645 | 17 |
|  |  |  |  | x |  |  |  |  |  |  | 17 |
|  |  | 2 | x |  |  | Japan | Mt. Kasatori, Ehime | N33°34.011’ | E132°56.012’ | 1425 | 17 |
|  |  |  |  | x |  |  |  |  |  |  | 17 |
| Indonemoura |  | 2 |  | x |  | Japan | Yada River, Hyogo | N35°24.518’ | E134°30.408’ | 420 | 17 |
|  |  |  |  |  | x |  |  |  |  |  | 17 |
|  |  | 2 |  | x |  | Japan | Yu River, Kagoshima | N31°12.499’ | E130°48.655’ | 60 | 17 |
|  |  |  |  |  |  |  |  |  |  |  | 17 |
|  |  | 1 | x |  |  | Japan | Yanagisawa-gawa river, Tochigi | N36°46.05’ | E139°22.27’ | 1640 | 18 |
|  |  | 4 | x |  |  | Japan | Ota kigawa river, Fukushima | N37°20.15’ | E139°47.07’ | 530 | 18 |
|  |  |  | x |  |  |  |  |  |  |  | 18 |
|  |  |  | x |  |  |  |  |  |  |  | 18 |
|  |  |  | x |  |  |  |  |  |  |  | 18 |
|  |  | 2 | x |  |  | Japan | Motoisago gawa river, Miyagi | N38°15.42’ | E140°34.40’ | 320 | 18 |
|  |  |  | x |  |  |  |  |  |  |  | 18 |
|  | I. scalprata | 1 |  | x |  | China | Neichao River, Guangxi | N23°29.664’ | E108°21.622’ | 220 | 19 |
|  | I. sp. n. | 2 | x |  |  | China | Fukumi-gawa, Guangxi | N33°50.952’ | E132°52.112’ | 515 | 20 |
|  |  |  |  | x |  |  |  |  |  |  | 20 |
|  | N. akagii | 2 | x |  |  | Japan | Takagamine, Kyoto | N35°03.369’ | E135°43.440’ | 150 | 21 |
|  |  |  |  | x |  |  |  |  |  |  | 21 |
|  | N. cf. cercispinosa | 2 | x |  |  | Japan | Jikibamachi, Ehime | N33°51.699’ | E132°49.560’ | 210 | 22 |
|  |  |  |  | x |  |  |  |  |  |  | 22 |
|  |  | 1 | x |  |  | Japan | Jikibamachi, Ehime | N33°51.579’ | E132°49.963’ | 280 | 22 |
|  |  | 2 | x |  |  | Japan | Toon, Ehime | N33°45.183’ | E132°59.512’ | 975 | 22 |
|  |  |  |  | x |  |  |  |  |  |  | 22 |
|  |  | 1 | x |  |  | Japan | Kawanouchi, Ehime | N33°45.386’ | E132°58.993’ | 700 | 22 |
|  |  | 1 |  | x |  | Japan | Kawanouchi, Ehime | N33°45.952’ | E132°58.756’ | 580 | 22 |
|  |  | 1 |  | x |  | Japan | Kawanogomachi, Ehime | N33°52.444’ | E132°52.030’ | 430 | 22 |
|  |  | 1 |  | x |  | Japan | Saijo, Ehime | N33°45.322’ | E133°09.436’ | 1450 | 22 |
|  |  | 2 | x |  |  | Japan | Mt. Kasatori, Ehime | N33°34.011’ | E132°56.012’ | 1425 | 22 |
|  |  |  | x |  |  |  |  |  |  |  | 22 |
|  |  | 1 |  | x |  | Japan | Karakai Falls, Ehime | N33°44.946’ | E132°58.878’ | 860 | 22 |
|  |  | 2 | x |  |  | Japan | Kuromori, Ehime | N33°45.524’ | E132°59.374’ | 990 | 22 |
|  |  |  |  | x |  |  |  |  |  |  | 22 |
|  |  | 2 | x |  |  | Japan | Yu River, Kagoshima | N31°12.255’ | E130°49.148’ | 105 | 22 |
|  |  |  |  | x |  |  |  |  |  |  | 22 |
|  |  | 2 | x |  |  | Japan | Nakatsu River, Fukushima | N37°40.475’ | E140°08.857’ | 800 | 23 |
|  |  |  |  | x |  |  |  |  |  |  | 23 |
|  | N. chinonis | 1 |  | x |  | Japan | Karakai Falls, Ehime | N33°44.912’ | E132°58.897’ | 870 | 24 |
|  |  | 1 |  | x |  | Japan | Ishizuchi, Ehime | N33°44.908’ | E133°09.056’ | 1450 | 24 |
|  |  | 1 |  | x |  | Japan | Nomuracho, Ehime | N33°28.508’ | E132°53.132’ | 1170 | 24 |
|  |  | 2 | x |  |  | Japan | Koide Stream, Kagawa | N34°07.172’ | E134°04.380’ | 400 | 24 |
|  |  |  |  | x |  |  |  |  |  |  | 24 |
|  |  | 1 | x |  |  | Japan | Yu River, Kagoshima | N31°12.255’ | E130°49.148’ | 105 | 24 |
|  |  | 1 |  | x |  | Japan | Hida Mts, Nagano | N36°33.645’ | E137°42.681’ | 1615 | 25 |
|  |  | 1 | x |  |  | Japan | Mt. Bandai, Fukushima | N37°36.271’ | E140°02.598’ | 990 | 25 |
|  | N. geei | 2 | x |  |  | South Korea | Seongbuk district, Anam-dong | N37°35.605’ | E127°01.682’ | 105 | 26 |
|  |  |  |  | x |  |  |  |  |  |  | 26 |
|  |  | 2 | x |  |  | China | Henan Providence | N35°42’ | E113°36’ | 1000 | 27 |
|  |  |  |  | x |  |  |  |  |  |  | 27 |
|  | N. longicercia | 2 | x |  |  | Japan | Ishizuchi, Ehime | N33°45.204’ | E133°08.857’ | 1500 | 28 |
|  |  |  |  | x |  |  |  |  |  |  | 28 |
| Nemoura |  | 2 | x |  |  | Japan | Ishizuchi, Ehime | N33°43.965’ | E133°07.236’ | 1060 | 28 |
|  |  |  |  | x |  |  |  |  |  |  | 28 |
|  |  | 2 |  | x |  | Japan | Karakai Falls, Ehime | N33°44.946’ | E132°58.878’ | 860 | 28 |
|  |  |  |  |  | x |  |  |  |  |  | 28 |
|  |  | 1 | x |  |  | Japan | Mt. Kamegamori, Kochi | N33°47.534’ | E133°11.708’ | 1675 | 28 |
|  |  | 2 | x |  |  | Japan | Narai River, Nagano | N35°58.194’ | E137°49.041’ | 940 | 28 |
|  |  |  |  |  | x |  |  |  |  |  | 28 |
|  | N. ovocercia | 1 | x |  |  | Japan | Mt. Chojamine, Nagano | N35°20.827’ | E137°37.857’ | 1555 | 29 |
|  | N. papilla | 2 | x |  |  | China | Wuliquan, Archang | N47°10’ | E119°55’ | 1035 | 30 |
|  |  |  |  | x |  |  |  |  |  |  | 30 |
|  | N. redimiculum | 1 | x |  |  | Japan | Sukawa, Akaya River, Gunma | N36°42.184’ | E138°54.629’ | 475 | 31 |
|  |  | 3 | x |  |  | Japan | Toyonakacho, Fudo, Kagawa | N34°09.049’ | E133°40.335’ | 75 | 31 |
|  |  |  |  | x |  |  |  |  |  |  | 31 |
|  |  |  |  |  | x |  |  |  |  |  | 31 |
|  | N. sanbena auct. n. | 2 | x |  |  | Japan | Koide Stream, Kagawa | N34°07.172’ | E134°04.380’ | 400 | 32 |
|  |  |  |  | x |  |  |  |  |  |  | 33 |
|  | N. securigera | 1 | x |  |  | China | Yuhuangshan, Henan | N34°3'34" | E111°3'23" | 1105 | 34 |
|  | N. yakushimana auct. n. | 2 | x |  |  | Japan | Tarumizu, Kagoshima | N31°30.256’ | E130°45.166’ | 345 | 35 |
|  |  |  | x |  |  |  |  |  |  |  | 35 |
|  | N. sp. n. 1 | 1 |  | x |  | Japan | Mt. Kamegamori, Kochi | N33°47.534’ | E133°11.708’ | 1675 | 36 |
|  |  | 2 | x |  |  | Japan | Mt. Kasatori, Ehime | N33°33.979’ | E132°56.136’ | 1390 | 36 |
|  |  |  |  | x |  |  |  |  |  |  | 36 |
|  | N. sp. n. 2 | 2 | x |  |  | Japan | Hachi, Betsuku, Hyogo | N35°23.051’ | E134°33.125’ | 755 | 37 |
|  |  |  |  | x |  |  |  |  |  |  | 37 |
|  | N. sp. n. 3 | 1 | x |  |  | Japan | Seki River, Niigata | N36°50.910’ | E138°08.958’ | 725 | 38 |
|  | N. sp. n. 4 | 1 | x |  |  | Japan | Yada River, Hyogo | N35°24.518’ | E134°30.408’ | 420 | 39 |
|  | N. sp. n.5 | 1 | x |  |  | China | Henan Providence | N31°45’ | E115°30’ | 500 | 40 |
|  | N. sp. n.6 | 1 | x |  |  | China | Sichuan | N30°54’ | E102°53’ | 3000 | 41 |
|  | N. sp. n.7 | 1 | x |  |  | China | Balangshan | N30°54’ | E102°53’ | 3000 | 42 |
|  | N. stratum | 1 | x |  |  | Japan | Shinmeigu, Nagano | N35°57.937’ | E137°48.583’ | 975 | 43 |
|  |  | 1 |  | x |  | Japan | Shirahone, Nagano | N36°09.049’ | E137°37.704’ | 1350 | 43 |
|  | N. taihangshana | 1 | x |  |  | China | Henan Providence | N33°55’ | E111°30’ | 1000 | 44 |
|  | N. transversospinosa | 1 | x |  |  | Japan | Kuromori, Ehime | N33°45.524’ | E132°59.374’ | 990 | 45 |
|  |  | 2 | x |  |  | Japan | Mt. Kotohira, Kagawa | N34°12.264’ | E133°46.795’ | 210 | 45 |
|  |  |  |  | x |  |  |  |  |  |  | 45 |
|  |  | 3 | x |  |  | Japan | Kuromori, Ehime | N33°45.524’ | E132°59.374’ | 990 | 45 |
|  |  |  | x |  |  |  |  |  |  |  | 45 |
|  |  |  |  | x |  |  |  |  |  |  | 45 |
|  | N. cf. hikosan | 1 | x |  |  | Japan | Kuromori, Ehime | N33°45.122’ | E132°59.371’ | 965 | 46 |
|  |  | 1 | x |  |  | Japan | Mt. Kasatori, Ehime | N33°33.979’ | E132°56.136’ | 1390 | 46 |
|  | N. fulva | 1 |  | x |  | Japan | Mt. Azuma, Fukushima | N37°45.252’ | E140°18.221’ | 815 | 47 |
|  |  | 2 | x |  |  | Japan | Narai River, Nagano | N35°58.194’ | E137°49.041’ | 940 | 47 |
|  |  |  |  | x |  |  |  |  |  |  | 47 |
|  | N. naraiensis | 2 | x |  |  | Japan | Ishizuchi, Ehime | N33°45.322’ | E133°09.436’ | 1450 | 48 |
|  |  |  |  | x |  |  |  |  |  |  | 48 |
|  | N. jilinensis | 2 | x |  |  | South Korea | Chiak Mts, Sillim-myeon, Wonju | N37°18.284 | E128°03.262 | 1025 | 49 |
|  |  |  |  |  | x |  |  |  |  |  | 49 |
|  | N. uenoi | 2 | x |  |  | Japan | Koide Stream, Kagawa | N34°07.172’ | E134°04.380’ | 400 | 50 |
|  |  |  |  | x |  |  |  |  |  |  | 50 |
|  |  | 1 | x |  |  | Japan | Akaya River, Gunma | N36°42.184’ | E138°54.629’ | 475 | 51 |
|  | N. shikokuensis auct. n. | 1 |  | x |  | Japan | Matsuyama, Ehime | N33°52.444’ | E132°52.030’ | 430 | 52 |
|  |  | 3 | x |  |  | Japan | Kuwaze, Kochi | N33°48.110’ | E133°16.785’ | 840 | 52 |
|  |  |  | x |  |  |  |  |  |  |  | 52 |
|  |  |  |  | x |  |  |  |  |  |  | 52 |
|  | N. speciosa auct. n. | 2 | x |  |  | Japan | Nakatsu River, Fukushima | N37°40.475’ | E140°08.857’ | 800 | 53 |
|  |  |  |  | x |  |  |  |  |  |  | 53 |
|  | P. kohnoae | 3 | x |  |  | Japan | Hida Mts, Nagano | N36°33.511’ | E137°43.096’ | 1475 | 54 |
|  |  |  | x |  |  |  |  |  |  |  | 54 |
|  |  |  | x |  |  |  |  |  |  |  | 54 |
|  |  | 4 | x |  |  | Japan | Nishi-Aduma Sky Valley, Yamagata | N37°45.102’ | E140°05.857’ | 1315 | 55 |
|  |  |  | x |  |  |  |  |  |  |  | 55 |
|  |  |  | x |  |  |  |  |  |  |  | 55 |
|  |  |  | x |  |  |  |  |  |  |  | 55 |
|  | P. orbiculata | 2 | x |  |  | Japan | Hachi, Betsuku, Hyogo | N35°23.051’ | E134°33.125’ | 755 | 56 |
|  |  |  |  | x |  |  |  |  |  |  | 56 |
|  |  | 2 | x |  |  | Japan | Mikuni, Niigata | N36°46.194’ | E138°48.928’ | 1075 | 56 |
|  |  |  |  |  | x |  |  |  |  |  | 56 |
| Protonemura |  | 2 | x |  |  | Japan | Ojiro-Atsuta, Hyogo | N35°23.669’ | E134°30.100’ | 650 | 56 |
|  |  |  |  | x |  |  |  |  |  |  | 56 |
|  | P. sp. n. | 2 | x |  |  | Japan | Ishizuchi, Ehime | N33°45.322’ | E133°09.436’ | 1450 | 57 |
|  |  |  |  | x |  |  |  |  |  |  | 57 |
|  | P. sp. n. 1 | 1 |  |  | x | Japan | Shomyo River, Toyama | N36°35.095’ | E137°29.256’ | 720 | 58 |
|  | P. sp. n. 2 | 2 | x |  |  | Japan | Mt. Adatara, Fukushima | N37°34.726’ | E140°14.946’ | 875 | 59 |
|  |  |  |  | x |  |  |  |  |  |  | 59 |
|  | P. sp. n. 3 | 1 | x |  |  | Japan | Hida Mts, Nagano | N36°33.645’ | E137°42.681’ | 1615 | 60 |
|  | P. sp. n. 5 | 1 |  |  | x | Japan | Mt. Sefuri, Saga | N33°25.665’ | E130°22.322’ | 895 | 61 |
