# Supplementary figures and images for: Molecular phylogeny and diversification timing of the Nemouridae family (Insecta, Plecoptera) in the Japanese Archipelago
Source: PLoS One. 2019 Jan 11;14(1):e0210269. doi: 10.1371/journal.pone.0210269 (PMC6329508; doi:10.1371/journal.pone.0210269)

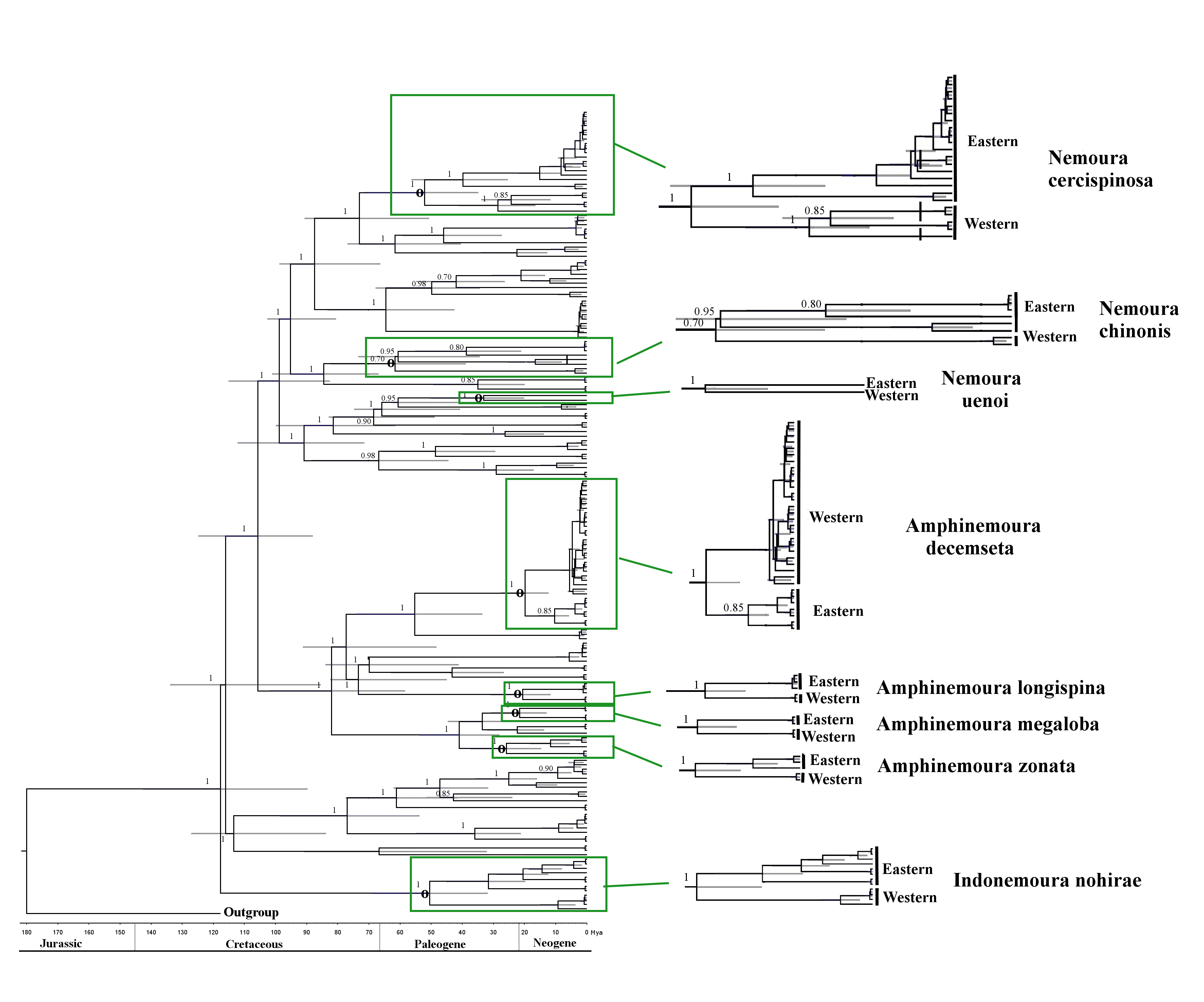

Supplement: S1 Fig — (TIFF) [file pone.0210269.s002.tiff]

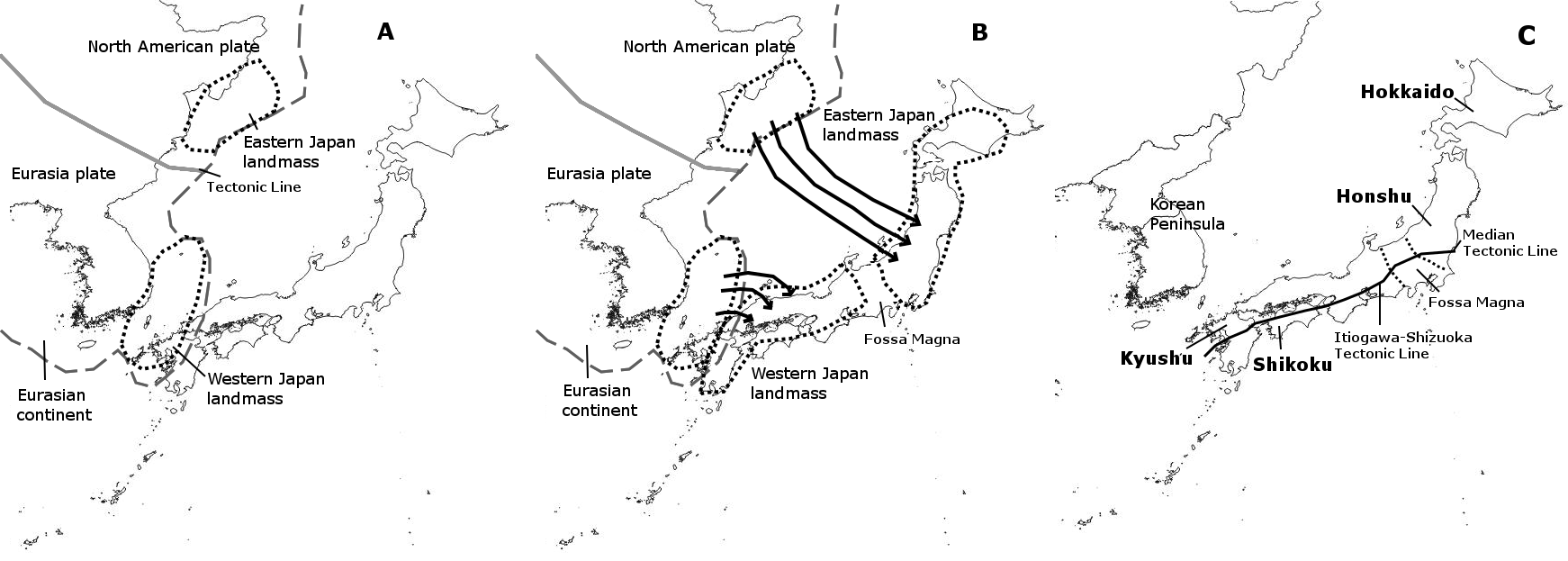

Supplement: S2 Fig — (A) Around 30 to 130 Ma, the Japanese landmasses were located in two major tectonic plates from the Eurasian continent. (B) Around 15 to 30 Ma, the Japanese landmasses began to separate from Eurasia and the North American Plates began to separate from the Eurasian continent, and remained separated by a sea zone called Fossa Magna—a geological event called double-door. (C) Current map of the Japanese Archipelago in East Asia, where the names of the four main Japanese islands and the two tectonic lines are shown. The maps was prepared using QGIS v 2.18 under the GNU free Documentation License with political boundaries from the Global Database of Administrative Areas (https://gadm.org/). (TIFF) [file pone.0210269.s003.tiff]
